# A nomogram for clinical estimation of acute biliary pancreatitis risk among patients with symptomatic gallstones: A retrospective case-control study

**DOI:** 10.3389/fcimb.2022.935927

**Published:** 2022-08-02

**Authors:** Xiaoyu Guo, Yilong Li, Hui Lin, Long Cheng, Zijian Huang, Zhitao Lin, Ning Mao, Bei Sun, Gang Wang, Qiushi Tang

**Affiliations:** ^1^ Department of Pancreatic and Biliary Surgery, The First Affiliated Hospital of Harbin Medical University, Harbin, China; ^2^ Department of Internal Medicine, The Second Affiliated Hospital of Shanxi Medical University, Taiyuan, China; ^3^ Chinese Journal of Practical Surgery, Chinese Medical University, Shenyang, China

**Keywords:** gallstones, acute biliary pancreatitis, predictors, nomogram, receiver operating characteristic curves

## Abstract

**Background/Purpose:**

Currently, there are no effective tools to accurately assess acute biliary pancreatitis (ABP) risk in patients with gallstones. This study aimed to develop an ABP risk nomogram in patients with symptomatic gallstones.

**Methods:**

We conducted a retrospective nested case-control study and data on 816 conservatively treated patients with symptomatic gallstones admitted to The First Affiliated Hospital of Harbin Medical University between January 6, 2007 and January 22, 2016 were retrospectively collected. We conducted a propensity-score matched (PSM) analysis based on follow-up time in a ratio of 1:4 between ABP group (n=65) and non-ABP group (n=260). These matched patients were randomly divided into study cohort (n=229) and validation cohort (n=96) according to a ratio of 7:3. In the study cohort, independent risk factors for ABP occurrence identified using Cox regression were included in nomogram. Nomogram performance and discrimination were assessed using the concordance index (C-index), area under the curve (AUC), calibration curve, decision curve analysis (DCA) and clinical impact curve (CIC). The model was also validated in the validation cohort.

**Results:**

Nomogram was based on 7 independent risk factors: age, diabetes history, gallbladder wall thickness, gallstone diameter, coexisting common bile duct (CBD) stones, direct bilirubin (DBIL), and white blood cell count (WBC). The C-index of nomogram was 0.888, and the 10-year AUCs of nomogram was 0.955. In the validation cohort, nomogram still had good discrimination (C-index, 0.857; 10-year AUC, 0.814). The calibration curve showed good homogeneity between the prediction by nomogram and the actual observation. DCA and CIC demonstrated that nomogram was clinically useful.

**Conclusions:**

The ABP risk nomogram incorporating 7 features is useful to predict ABP risk in symptomatic gallstone patients.

## Introduction

Acute pancreatitis (AP), as a common acute abdominal condition, is an inflammatory disorder of the pancreas accompanied by potentially severe local or systemic complications and high mortality (Guo et al., 2019). As a leading cause of admission to the hospital for gastrointestinal disorders worldwide, AP is characterized by the main clinical feature of autodigestion of the pancreas, sometimes accompanied by multiple organ dysfunction. There are various etiological factors capable of inducing an acute attack of AP, such as gallstones, alcohol misuse, smoking, drug use, genetic factors, and tumors (Lowenfels et al., 2000). According to these factors, AP is frequently divided into acute biliary pancreatitis (ABP), acute alcoholic pancreatitis, acute hyperlipidemic pancreatitis, acute idiopathic pancreatitis and so forth (Nauck, 2013).

ABP is recognized as the leading type of AP worldwide, accounting for 35-60% of AP cases, with a reported mortality rate ranging from 5% to 20%. The pathogenesis of ABP might be associated with passage of small gallbladder stones or biliary sludge through the ampulla of Vater and other factors, such as anatomical variations, iatrogenic factors including surgical operation and endoscopic retrograde cholangiopancreatography (ERCP), ampullary carcinoma, and pancreatic head carcinoma (Ridtitid et al., 2019). Of all these risk factors, gallstones are still the main cause of ABP. Studies suggest that ABP is frequently the first symptom of gallstone disease in approximately 40% of patients without a preceding episode of biliary colic (van Erpecum, 2006). Clinical data and experience also show that not all patients with symptomatic gallstones will eventually develop ABP, which results in an arduous challenge and several questions: Which patients with gallstones are more likely to develop ABP? What are the related risk factors for ABP? How can clinicians accurately predict the occurrence of ABP in patients with gallstones and take timely preventive measures?

Some scholars have provided a nomogram combining CT and clinical features for early diagnosis of ABP in admission, however, as for early prevention of ABP, the development of a prediction model for ABP among patients with symptomatic gallstones is still desirable (Zver et al., 2022). Of all the available models, a nomogram can provide an individualized, evidence-based, highly accurate risk estimation. And nomograms are easy to use and can facilitate management-related decision making (Balachandran et al., 2015).

## Method

### Patients

Between January 6, 2007, and January 22, 2016, data on inpatients were retrospectively collected from the First Affiliated Hospital of Harbin Medical University. The patients who meet inclusion criteria were mainly diagnosed with symptomatic gallstones during hospitalization and most patients were admitted to the hospital for acute abdominal pain. All patients in this study were treated conservatively after admission. The exclusion criteria mainly included (1) incomplete medical record data and serum test and imaging examination results, (2) patients used to undergo any surgical and endoscopic treatment such as cholecystectomy and ERCP (3) ambiguous diagnosis, (4) other concomitant major diseases that would interfere with the study, such as heart failure, renal failure, and multiple organ dysfunction syndrome (MODS), (5) female patients during pregnancy or lactation. These patients were followed from the day of discharge until the last documented follow-up. The study was approved by the Ethics Committee of First Affiliated Hospital of Harbin Medical University (ethics board approval number: ChiCTR1800016492). All the patients’ data were used for only research. The study did not affect the treatment of patients. The primary endpoint was ABP excluding other types of AP such as hypertriglyceridemic and alcoholic acute pancreatitis. Non-ABP controls were matched to ABP cases by a ratio of 4:1 using a propensity-score matched (PSM) analysis based on follow-up time. Among ABP cases and non-ABP controls, some patients were randomly divided into the study cohort for nomogram development; the others formed the validation cohort to confirm the model’s performance.

### ABP diagnostic criteria

The ABP diagnostic criteria were as follows: (1) having gallstones confirmed by abdominal ultrasound, CT, MRCP or other imaging examination; (2) having two or more of the following laboratory examination indicators: ①alkaline phosphatase (AKP)>125 U/L, ②alanine transaminase (ALT)>150 U/L, ③total bilirubin (TBIL)>2.3 mg/dl, and ④gamma-glutamyl transferase (GGT)>40 U/L; (3) conforming to the diagnostic criteria of AP and having at least 2 of the following 3 clinical characteristics: ① abdominal pain consistent with AP; ② serum amylase and/or lipase activity at least 3 times higher than the upper limit of normal; ③ abdominal imaging examination consistent with the imaging changes associated with AP; and (4) no other causes of abnormality of serum amylase and lipase and liver function test (Zhao, 2002; Zhu and Lin, 2012; Coffey et al., 2013; Surlin et al., 2014).

### Clinicopathologic variables

The clinicopathologic variables in this study are reported in **Table 1**, and these variables were recorded before diagnosis of ABP. The imaging data included gallbladder size, thickness of the gallbladder wall, number of gallstones, diameter of gallstones, gallstone morphology, coexisting common bile duct (CBD) stone, and diameter of the bile duct. The normal size of the adult gallbladder was defined as 7-10 cm in length and 3-4 cm in transverse diameter (John et al., 2017). A gallbladder with a size differing from this criteria was seen as abnormal. The gallbladder wall thickness, as an objective marker of cholecystitis severity and laparoscopic cholecystectomy (LC) complexity, was divided into two groups: ≤3 mm (normal) and >3 mm (Kokoroskos et al., 2020). The characteristics of gallstones were represented by the number of gallstones, the diameter of gallstones and the gallstone shapes. The number of gallstones was divided into two groups: solitary gallstones and multiple gallstones (≥2). According to the gallstone shapes shown by imaging examinations, the shapes were classified into spherical stones, sand-like stones and irregular stones. Considering that smaller gallstones were frequently seen in patients with AP, especially stone sizes <3 mm, the diameter of gallstones was separated into three groups: <3 mm, 3~10 mm and >10 mm, and the minimum diameter of gallstones was recorded in case of multiple gallstones (Gonzalez et al., 2012). During the imaging examination, two experienced radiologists independently evaluated all imaging data. Any controversies in imaging findings between radiologists were settled by discussion, and a final standard radiologic report on each patient was generated. At present, although abdominal ultrasound is still the most common method of screening for gallstones and bile duct stones, due to intestinal gas interference, the diagnostic value is always limited (Soyer et al., 2013). CT is clinically more valuable than ultrasound in diagnosing gallstones and bile duct stones, but it is easily affected by stone composition and density, especially low-density stones with cholesterol as the main component (Chan et al., 2006). In contrast, MRCP’s sensitivity, specificity, and diagnostic accordance rate in the diagnosis of gallstones are higher than those of ultrasound and CT (Griffin et al., 2012). Therefore, the imaging results of MRCP were recorded preferentially in this study.

**Table 1:** Characteristics of patients in the study and validation cohorts.

Variables	Study cohort(n=229)	Validation cohort(n=96)	*P*-value
Age (year)			
<50	104 (45.4%)	50 (52.1%)	
≥50	125 (54.6)	46 (47.9%)	0.272
Sex			
Female	148 (64.6%)	52 (54.2%)	
Male	81 (35.4%)	44 (45.8%)	0.77
Alcoholic history			
No	192 (83.8%)	75 (78.1%)	
Yes	37 (16.2%)	21 (21.9%)	0.219
Smoking history			
No	190 (83.0%)	79 (82.3%)	
Yes	39 (17.0%)	17 (17.7%)	0.883
Diabetes history			
No	172 (75.1%)	67 (69.8%)	
Yes	57 (24.9%)	29 (30.2%)	0.321
Gallbladder size			
Normal	167 (72.9%)	62 (64.6%)	
Abnormal	62 (27.1%)	34 (35.4%)	0.133
Gallbladder wall thickness (mm)			
≤3	137 (59.8%)	58 (60.4%)	
>3	92 (40.2%)	38 (39.6%)	0.921
Gallstone number			
1	33	14	
≥2	196	82	0.968
Gallstone diameter (mm)			
<3mm	49 (21.4%)	18 (18.8%)	
3-10mm	112 (48.9%)	54 (56.3%)	
>10mm	68 (29.7%)	24 (24.9%)	0.479
Gallstone shape			
Sphere	121 (52.8%)	66 (68.8%)	
Irregular	53 (23.1%)	17 (17.7%)	
Sand-like	55 (24.1%)	13 (13.5%)	0.240
Bile duct stones			
No	175 (76.4%)	76 (79.2%)	
Yes	54 (23.6%)	20 (20.8%)	0.590
Diameter of CBD (mm)			
≤10	193 (84.3%)	84 (87.5%)	
>10	36 (15.7%)	12 (12.5%)	0.455
ALT (U/L)			
<150U/L	180 (78.6%)	83 (86.5%)	
≥150U/L	49 (21.4%)	13 (13.5%)	0.100
AST (U/L)			
<53.6U/L	161 (70.3%)	72 (75.0%)	
≥53.6U/L	68 (29.7%)	24 (25.0%)	0.391
AST/ALT			
<1.0	145 (63.3%)	50 (52.1%)	
≥1.0	84 (36.7%)	46 (47.9%)	0.059
GGT (U/L)			
<150	139 (60.7%)	66 (68.8%)	
≥150	90 (39.3%)	30 (31.2%)	0.170
AKP (U/L)			
<125	162 (70.7%)	65 (67.7%)	
≥125	67 (29.3%)	31 (32.3%)	0.587
TBIL (mg/dL)			
<1.4	151 (65.9%)	67 (69.8%)	
≥1.4	78 (34.1%)	29 (30.2%)	0.500
IBIL (mg/dL)			
<0.8	182 (79.5%)	80 (83.3%)	
≥0.8	47 (20.5%)	16 (16.7%)	0.422
DBIL (mg/dL)			
<1.0	196 (85.6%)	83 (86.5%)	
≥1.0	33 (14.4%)	13 (13.5%)	0.838
WBC (×10^9^/L)			
<10	164 (71.6%)	68 (70.8%)	
≥10	65 (28.4%)	28 (29.2%)	0.887
GRAN% (%)			
<80	147 (64.2%)	59 (61.5%)	
≥80	82 (35.8%)	37 (38.5%)	0.641

P<0.05 was statistically significant.

AKP, Alkaline phosphatase; ALT, Alanine transaminase; AST, Aspartate transaminase; CBD, Common bile duct; DBIL, Direct bilirubin; GGT, Gamma-glutamyl transferase; GRAN%, Granulocyte%; IBIL, Indirect bilirubin; TBIL, Total bilirubin; WBC, White blood cell count.

### Statistical analysis

To minimize bias between the ABP and non-ABP groups, we conducted a propensity-score matched (PSM) analysis in a ratio of 1:4. After samples matching, these samples were randomly divided into study cohort and validation cohort according to the ratio of 7:3. Frequency (ratio) was utilized to describe the characteristics of categorical variables, and comparisons between the two cohorts were performed using chi-square tests. Then the data in the study cohort were used to establish a model and the data in validation set were applied to evaluate the efficacy of the model. Based on the data in the study cohort, univariable Cox proportional hazards analysis was performed for each variable. P-values of the variables were calculated based on the univariable Cox proportional hazards regression model. The variables with p-values less than 0.05 were included in a multivariable Cox proportional hazards regression model. Then, the factors with p-value less than 0.05 were included in the prediction model to establish nomogram. In the nomogram, the sum of these points, plotted on the “total points” line, corresponded to the prediction of 10-year ABP occurrence-free rates in patients with symptomatic gallstones. Receiver operating characteristic curve (ROC) analysis and Harrell’s concordance index (C-index) were used to assess the discrimination of the model, and a calibration plot was used for internal verification. Decision curve analysis (DCA) and clinical impact curve (CIC) were utilized to evaluate the clinical application value of the model. Kaplan–Meier cumulative hazard analysis was used to estimate the risk of being diagnosed as ABP during the follow-up period. All analyses were performed using SPSS (22.0 IBM, Armonk, NY, USA) and R (version 4.2.1) software.

## Results

### Clinicopathologic characteristics and univariate analysis results

**Figure 1:**
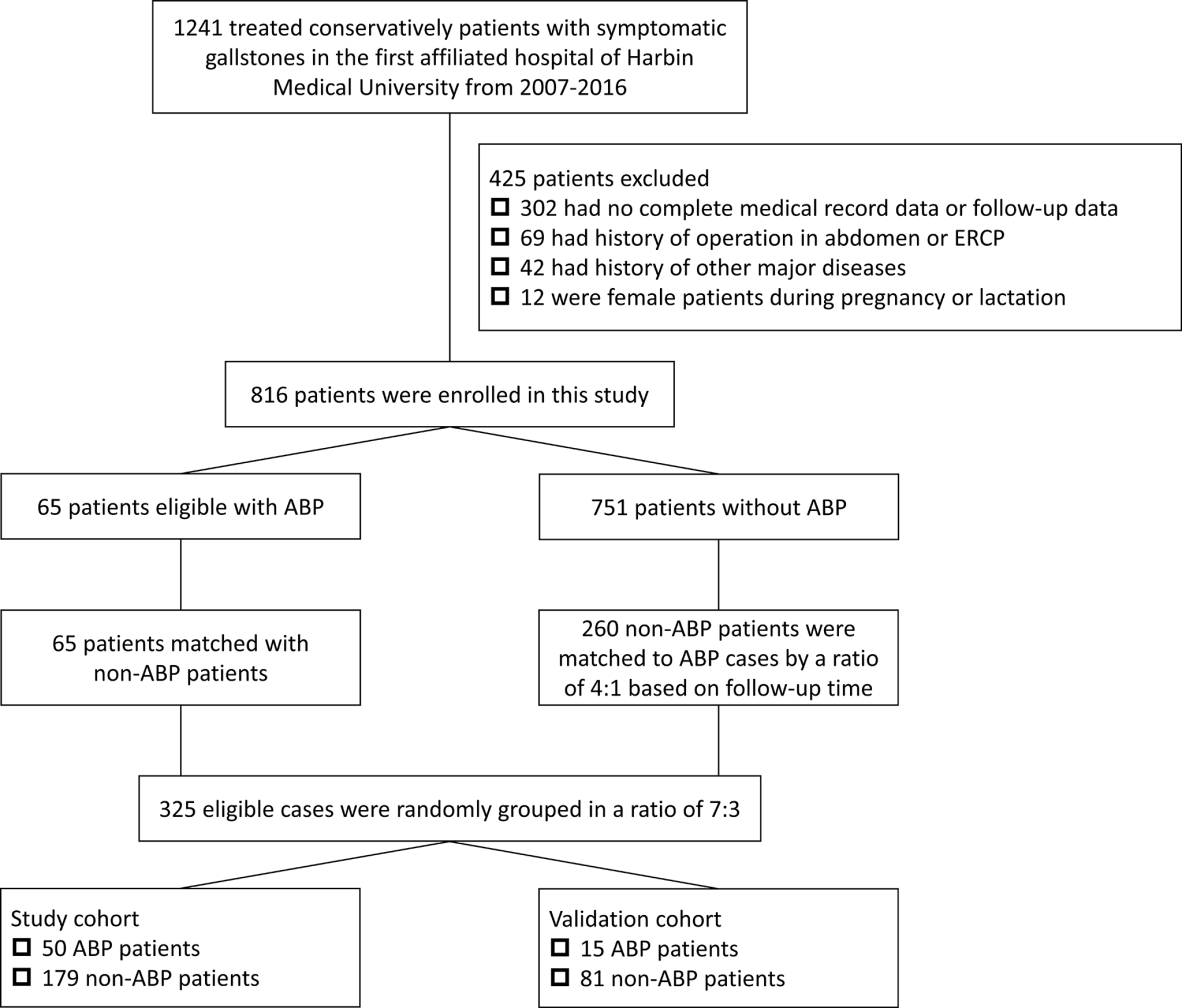
Flow chart of the study.

**Figure 2:**
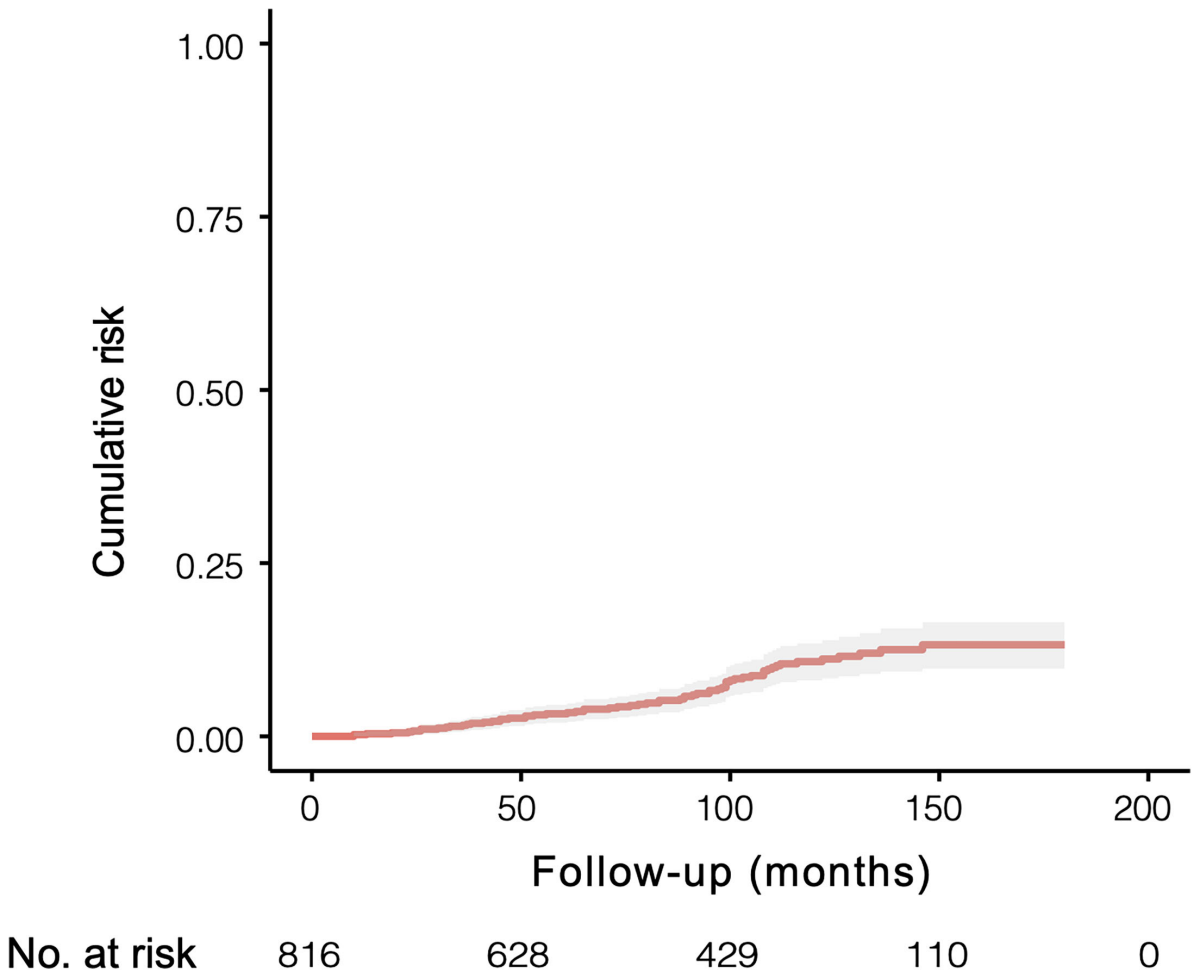
Cumulative risk of being diagnosed as ABP in 816 individuals with symptomatic gallstones and followed for a median of 89 months [IQR 45.8-130.0 months].

In this study, after exclusion, 816 patients with gallstones who met the inclusion criteria were finally enrolled. We conducted a propensity-score matched (PSM) analysis based on follow-up time in a ratio of 1:4 between ABP group (n=65) and non-ABP group (n=260). Then matched patients were randomly divided into study cohort (n=229) and validation cohort (n=96) according to a ratio of 7:3 (**Figure 1**). The median follow-up time after discharge was 89 months [IQR 45.8-130.0 months]. The 10-year cumulative risks of being diagnosed as ABP were 12.59% (95% CI 9.41% to 15.66%) during the follow-up, and this risk appeared to continue in subsequent years (**Figure 2**).

The clinicopathologic characteristics of the patients are listed in **Table 1**. The baseline clinicopathologic data were similar between the study and validation cohorts. The univariate cox analysis result of the study cohort showed that age, sex, alcoholic history, diabetes history, gallbladder wall thickness, gallstone number, gallstone diameter, gallstone shape, coexisting CBD stone, GGT, AKP, TBIL, direct bilirubin (DBIL), white blood cell count (WBC) and granulocyte% (GRAN%) were significantly different between the ABP group and the non-ABP group (p <0.05) (**Table 2**).

**Table 2:** Univariate and multivariate Cox analysis of the study cohort.

Variables	Univariate analysisHR (95% CI)	*P*-value	Multivariate analysisHR (95% CI)	*P*-value
Age (year)				
≥50 vs. <50	5.479 (2.545-11.797)	**<0.001**	3.491 (1.514-8.047)	**0.003**
Sex				
Male vs. Female	2.844 (1.575-5.135)	**0.001**	1.250 (0.537-2.911)	0.604
Alcoholic history				
Yes vs. No	1.955 (1.012-3.778)	**0.046**	0.917 (0.371-2.267)	0.851
Smoking history				
Yes vs. No	1.941 (1.001-3.764)	0.05		
Diabetes history				
Yes vs. No	5.378 (2.978-9.712)	**<0.001**	4.585 (1.926-10.914)	**0.001**
Gallbladder size				
Abnormal vs. Normal	0.732 (0.363-1.476)	0.384		
Gallbladder wall thickness (mm)				
>3 vs. ≤3	0.308 (0.152-0.622)	**0.001**	0.195 (0.079-0.480)	**<0.001**
Gallstone number				
≥2 vs. 1	4.394 (1.065-18.126)	**0.041**	1.559 (0.334-7.270)	0.572
Gallstone diameter (mm)				
3-10mm vs. <3mm	0.267 (0.112-0.636)	**0.003**	0.311 (0.105-0.924)	**0.035**
>10mm vs. <3mm	0.402 (0.216-0.749)	**0.004**	0.248 (0.094-0.655)	**0.005**
Gallstone shape				
Irregular vs. Sphere	0.762 (0.339-1.713)	0.511	0.842 (0.322-2.202)	0.726
Sand-like vs. Sphere	2.086 (1.105-3.937)	**0.023**	1.018 (0.454-2.285)	0.965
Coexisting CBD stones				
Yes vs. No	3.522 (1.981-6.262)	**<0.001**	2.382 (1.177-4.821)	**0.016**
Diameter of CBD (mm)				
>10 vs. ≤10	0.397 (.292-1.630)	0.397		
ALT (U/L)				
≥150U/L vs. <150U/L	1.809 (0.988-3.312)	0.550		
AST (U/L)				
≥53.6U/L vs. <53.6U/L	1.528 (0.857-2.725)	0.151		
AST/ALT				
≥1.0 vs. <1.0	0.979 (0.540-1.776)	0.944		
GGT (U/L)				
≥150 vs. <150	2.428 (1.323-4.458)	**0.004**	0.436 (0.143-1.335)	0.146
AKP (U/L)				
≥125 vs. <125	1.695 (0.949-3.029)	0.075		
TBIL (mg/dL)				
≥1.4 vs. <1.4	2.273 (1.267-4.078)	**0.006**	1.049 (0.380-2.893)	0.926
IBIL (mg/dL)				
≥0.8 vs. <0.8	0.810 (0.378-1.735)	0.588		
DBIL (mg/dL)				
≥1.0 vs. <1.0	2.214 (1.095-4.121)	**0.026**	4.867 (1.734-13.660)	**0.003**
WBC (×10^9^/L)				
≥10 vs. <10	5.494 (2.998-10.066)	**<0.001**	3.628 (1.397-9.427)	**0.008**
GRAN% (%)				
≥80 vs. <80	4.033 (2.198-7.398)	**<0.001**	1.717 (0.728-4.051)	0.217

P<0.05 was statistically significant.

AKP, Alkaline phosphatase; ALT, Alanine transaminase; AST, Aspartate transaminase; CBD, Common bile duct; DBIL, Direct bilirubin; GGT, Gamma-glutamyl transferase; GRAN%, Granulocyte%; IBIL, Indirect bilirubin; TBIL, Total bilirubin; WBC, White blood cell count. ‘bold values’ means statistical significant values (<0.05).

### Multivariate analysis result and establishment of an ABP-predicting nomogram

**Figure 3:**
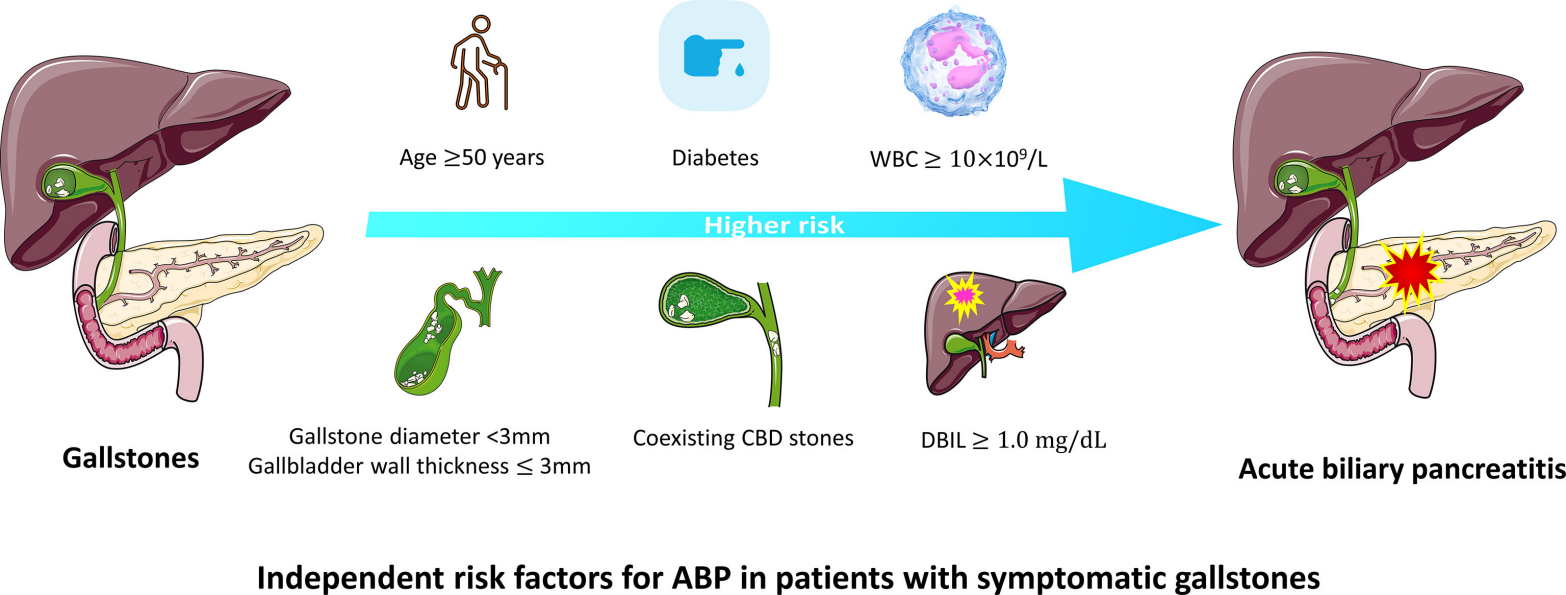
Independent risk factors for ABP in patients with gallstones.

**Figure 4:**
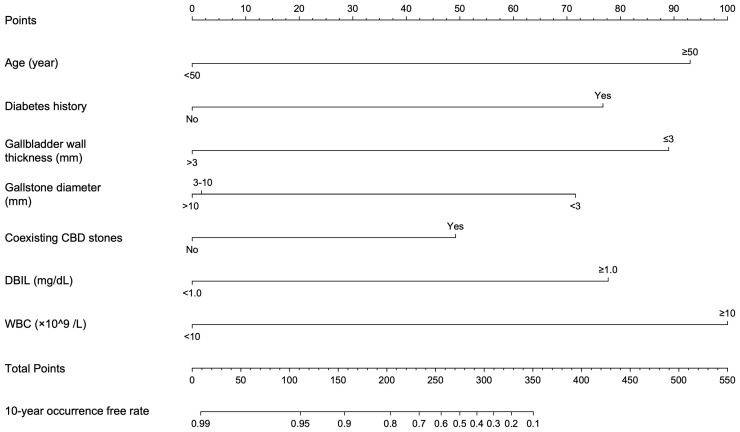
Nomogram conducted by Cox regression, including age, diabetes history, gallbladder wall thickness, gallstone diameter, coexisting CBD stones, DBIL and WBC in patients with symptomatic gallstones.

The significant factors obtained in the univariate cox analysis results were included in the multivariate cox regression model to analyze whether each factor was an independent risk factor for inducing ABP. In the multivariate analysis, with results reported as HRs (95% CIs), age >50 years (3.491 [1.514-8.047]), diabetes history (4.585 [1.926-10.914]), gallbladder wall thickness >3 mm (0.195 [0.079-0.480]), gallstone diameter (3-10 vs <3 mm, 0.311 [0.105-0.924], >10 mm vs <3mm, 0.248 [0.094-0.655]), coexisting CBD stone (2.382 [1.177-4.821]), DBIL >1.0 mg/dL (4.867 [1.734-13.660]) and WBC >10×109 (3.628 [1.397-9.427]) were independently associated with ABP (**Table 2**; **Figure 3**). These independently associated risk factors were utilized to make an ABP risk estimation nomogram based on 10-year ABP-occurrence free rate (**Figure 4**).

### Validation and effect evaluation of the nomogram

**Figure 5:**
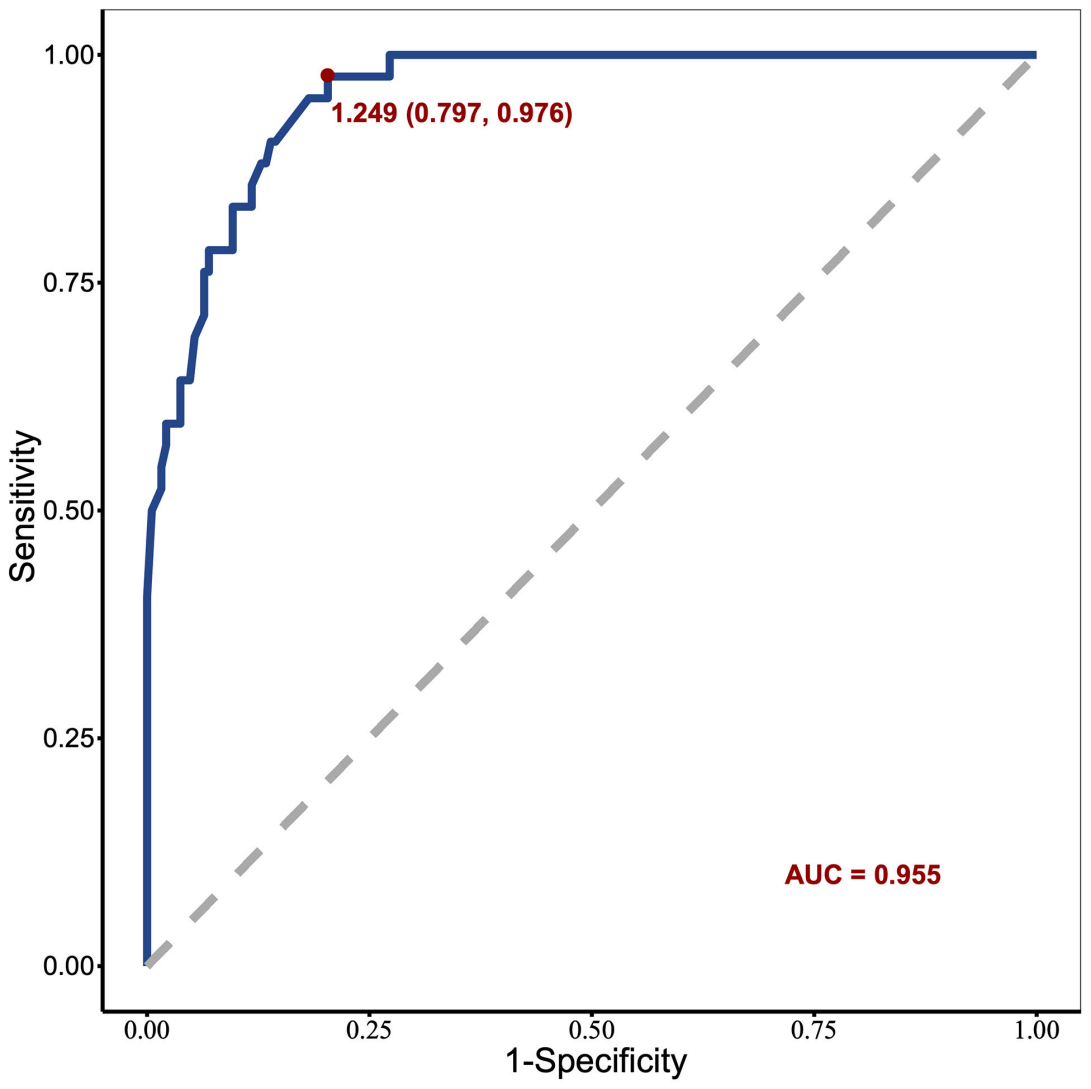
Receiver operating characteristic curve of the nomogram in the study cohort.

**Figure 6:**
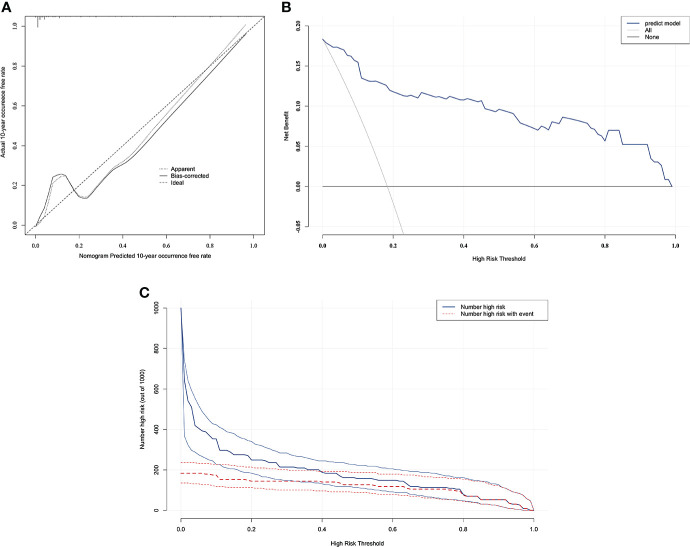
The calibration curve and results of the DCA and the CIC analysis of the nomogram in the validation cohort. **(A)** Calibration curves represent the difference between the actual prediction and the ideal perfect prediction (45◦ line). **(B)** The DCA curve of the nomogram for predicting ABP. It revealed that the nomogram could obtain a greater net benefit than either the “treat all” or the “treat none” strategy. **(C)** The CIC curve of the nomogram for predicting ABP. The solid blue line (Number high risk) represents the number of ABP patients predicted using the nomogram at each threshold probability; the dotted red line (number high risk with event) represents the number of true-positive ABP patients at each threshold probability.

The resulting model was internally validated based on validation cohort. The performance of the nomogram was measured by ROC curve (**Figure 5**). The C-index of nomogram was 0.888, and the 10-year AUCs of nomogram were 0.955. In the validation cohort, nomogram still had good discrimination (C-index, 0.857; 10-year AUC, 0.814). Based on cutoff value of ROC in study cohort (1.249), the sensitivity and specificity of prediction model in validation cohort were 73.3% and 79.0% (97.6% and 79.7% in study cohort) (**Table 3**). The calibration curve showed good homogeneity between the prediction by nomogram and the actual observation (**Figure 6**). The decision curve analysis for the ABP incidence risk nomogram is also presented in **Figure 6**. The DCA demonstrated that the prediction model could provide great net benefit and make valuable and profitable judgements. The CIC result showed that the number of patients who were at high risk (the number of ABP patients predicted using the nomogram) was well matched with the number of patients who were at high risk with the event (the number of truly-diagnosed ABP patients) (**Figure 6**).

**Table 3:** Accuracy of prediction model in validation cohorts.

Prediction	Actual observation	
	ABP	Non-ABP
ABP	11	17
Non-ABP	4	64

Sensitivity of model: 73.3% (11/15).

Specificity of model: 79.0% (64/81)/

### Performance of the nomogram

As seen in nomogram, selected predictors were assigned with a score according to the value in the nomogram based on the established prediction model. Then a vertical line perpendicular to the point axis was drawn from this point. The intersection point on the point axis represented the score under the determined value of the predictor. For example, for a 55-year-old patient with gallstones and a history of diabetes, abdominal ultrasound suggested that the thickness of gallbladder wall was 3 mm, the diameter of gallstones was 3~5mm and there was no CBD stone. The laboratory examination results were as follows: DBIL 1.0 mg/dL; WBC 9×109/L. The 10-year occurrence free rate of ABP for this patient can be calculated as 30%. Considering the worse prognosis of elderly patients with acute pancreatitis, therefore, we recommend that the patient receive medical check-ups regularly and even undergo cholecystectomy if necessary to prevent occurrence of ABP.

## Discussion

AP is characterized by acute onset, rapid progression and a high likelihood of developing severe acute pancreatitis (SAP), with severe complications and a high mortality rate of 30% (Guo et al., 2019). Gallstones are still one of the key causative factors of AP, and in the present study, approximately 20% of gallstone patients in hospitals were diagnosed with ABP (Zhu and Lin, 2012). Therefore, it is clinically important for clinicians to prevent gallstone patients from developing AP and SAP. Our study also suggests that risk factors, including age, diabetes history, gallbladder wall thickness, gallstone diameter, coexisting CBD stone, DBIL and WBC, are significantly associated with the incidence of ABP.

In line with the current literature, our data regarding incidence of ABP indicate that elderly patients with gallstones had higher risk of ABP during follow-up (HR: 3.491, 95% CI: 1.514~8.047). The relation between advanced age and incidence of AP is not surprising. For instance, most studies indicate that the mean age of the first AP attack is 60 years and with increasing age, the incidence and mortality of AP are also increasing, and there have been studies indicating a correlation between age and mortality as an independent risk factor (Spanier BW and Bruno, 2008; Beard et al., 2016). Relevant studies have demonstrated that although some controversy exists, diabetic patients are generally thought to have a twofold to threefold increased risk of cholesterol gallstones. Moreover, owing to poor anti-infection ability and immunity, when patients with diabetes suffer cholecystitis, they are prone to serious biliary tract infection and even other severe complications, such as gallbladder abscess, gangrene, and perforation (Ransohoff et al., 1987). In this study, diabetes history is an important risk factor for ABP (HR: 4.585, 95% CI: 1.926~10.914). We speculate that diabetes is more likely to be associated with biliary tract infection and that biliary tract function is worse in patients with diabetes than in the normal population. Therefore, gallstone incarceration is more likely to occur during the downward movement of gallstones, thus inducing ABP.

Among the factors related to gallstones and the biliary tract, this study showed that the thickness of the gallbladder wall, gallstone diameter, and coexisting CBD stone were all significant risk factors for ABP in patients with gallstones. The risk of ABP in the normal gallbladder group was significantly higher than that in the abnormal gallbladder group. In addition, gallbladder wall thickness ≤ 3 mm was a risk factor for ABP (in comparison, the HR of the thickness of > 3 mm was 0.195, 95% CI: 0.079~0.480); that is, the risk of ABP in patients with a thickness of the gallbladder wall ≤ 3 mm was 5.13 times as high as that of patients with a thickness of > 3 mm. The reason might be that when the gallbladder wall thickness is normal, the gallbladder’s contraction function is relatively good (Yamada and Yamada, 2001). Therefore, when the gallbladder contracts, the gallbladder tube is able to normally expand, thus making gallstones easily discharged into the CBD and inducing ABP. In contrast, long-term inflammation frequently leads to a thicker gallbladder wall and relatively poor gallbladder contraction function, and the gallbladder duct has difficulty expanding (Stads et al., 2007). Therefore, gallstones are difficult to discharge and are likely to remain in the gallbladder, making the risk of ABP relatively lower. In the gallstone size analysis, patients were divided into three groups according to stone diameter: < 3 mm, 3-10 mm and >10 mm, and patients with gallstone diameters < 3 mm were 3.22 times more likely to develop ABP than those with diameters 3-10 mm and 4.03 times more likely to develop ABP than those with diameters >10 mm. The reason may be that when the gallbladder contracts, stones with smaller diameters are more likely to enter the common channel, and they easily cause channel blockage and finally induce ABP. In contrast, larger diameter stones tend to be incarcerated in the gallbladder neck and do not easily enter the common channel. Therefore, the risk of inducing ABP is relatively reduced (van Geenen et al., 2010). Coexisting CBD stone were also an essential risk factor for ABP in gallstone patients (HR: 2.382, 95% CI: 1.177~4.821). Our analysis showed that the risk of ABP in patients with gallstones and bile duct stones was about twice as high as that in patients with only gallstones. The reason is possibly that compared with gallstones, bile duct stones are more likely to cause duodenal papillary edema or stenosis, especially in the ampullary segment and the lower CBD, which are more likely to result in ABP than the upper CBD (Lee et al., 2018).

In terms of biochemical indicators commonly used in clinical practice, combined with multivariate cox regression analysis, we demonstrated that DBIL and WBC were all important and independent risk factors for ABP development in patients with gallstones. Among them, the abnormal level of DBIL reflects the degree of bile duct obstruction during the pathogenesis of ABP and the degree of hepatocyte injury caused by bile reflux (Surlin et al., 2014). Moreover, the level of WBC is able to reflect the severity of biliary tract infection during ABP (Lankisch et al., 2015). In general, when patients with gallstones suffer severe obstruction and infection of the biliary tract, their risk of concurrent ABP is obviously higher, and these biochemical indicators have good prediction value for ABP.

There are no guidelines that indicate which patient to offer a cholecystectomy or conservative treatment. Therefore, the indication to perform a cholecystectomy always lies within the surgeons’ preference leading to variations in practice and consequently unnecessary cholecystectomies. Some findings show that asymptomatic gallstone patients should not undergo prophylactic cholecystectomy. In a study by Gracie et al., only 18% of asymptomatic gallstone carriers developed biliary pain or a gallstone complication during 15-year follow-up (Gracie and Ransohoff, 1982). Furthermore, symptomatic complicated gallstone patients, especially ones with mild-to-moderate acute cholecystitis, common bile duct stones, or mild biliary pancreatitis, are now recommended for same admission cholecystectomy as opposed to delayed cholecystectomy in previous guidelines (MP., 2018).

However, for symptomatic uncomplicated gallstone patients, who are primarily involved in this study, whether and when cholecystectomy should be performed remains controversial. Most studies recommend that cholecystectomy is the therapy of the first choice for patients with uncomplicated symptomatic gallstone disease. Many patients have had an unnecessary cholecystectomy with associated risks of complications and unnecessary healthcare expenses, and some studies indicated that up to 33% of patients do not experience relief of their abdominal symptoms, despite cholecystectomy (Lamberts et al., 2013). Moreover, some patients with suggested uncomplicated symptomatic gallstone disease should be treated conservatively because of a high risk of persistent symptoms or suboptimal benefit of cholecystectomy. However, from the perspective of ABP prevention, there are still no uniform guidelines for choosing the optimal timing of cholecystectomy. Although some patients are at high risk of ABP in clinical practice, they may still choose conservative treatment and refuse effective surgical treatment for some reasons. All these above may increase the potential risk of ABP in patients with gallstones. In this study, most of the samples are asymptomatic uncomplicated patients with gallstones. Therefore, the nomogram can be used to identify these patients’ potential risk for ABP through commonly used clinical indicators and to help clinicians make better clinical decisions on the optimal timing of cholecystectomy. Moreover, it is also beneficial to encourage patients to avoid risk factors for ABP and receive a medical check-up regularly. Finally, it is worth noting that in this study, decision curves indicate that when the risk of ABP in patients is greater than 10%, carrying effective intervention will bring the population positive overall benefit. However, the intervention may include regular follow-up, regular medical check-ups, endoscopic treatment and surgical treatment. According to patients’ different risks for ABP, which intervention effectively prevents the occurrence of ABP remains to be further studied.

Our study had some limitations. First, this analysis was based on data from a single institution; it is necessary to validate the results in other centers. Second, there are still many risk factors affecting the incidence of ABP in patients with gallstones. Due to limited data, the risk factors selected in this study were not complete. Some potential risk factors of patients, such as body mass index (BMI) and blood lipids, were not included in the study, and more risk factors should be included in this study to further improve the accuracy of the prediction model. Finally, although the nomogram is more convenient than the traditional statistical model, it is no denying that there are still some limitations in the actual application. In the future, we will put the scoring system on a website or an app for use on a smart phone for surgeons in the hospital, and the score could automatically calculate results online.

## Conclusion

By combining seven clinical risk factors for ABP in symptomatic gallstone patients, a nomogram was constructed. The model provides an accurate and optimal estimation of ABP risk in patients with symptomatic gallstones. The nomogram provides an effective tool for quantitative clinical assessment of risks and benefits, which is conducive to the early prevention and treatment of ABP in patients with symptomatic gallstones. This model could also help clinicians and patients make scientific clinical decisions to maximize the clinical benefits of patients. 

## Data availability statement

The raw data supporting the conclusions of this article will be made available by the authors, without undue reservation.

## Ethics statement

The studies involving human participants were reviewed and approved by the Ethics Committee of First Affiliated Hospital of Harbin Medical University (ethics board approval number: ChiCTR1800016492). The patients/participants provided their written informed consent to participate in this study.

## Author contributions

XG, YL and HL contributed equally to this article. XG, YL and HL participated in the design of the study and drafted the manuscript. LC, ZH, ZL and NM participated in patients follow-up and data collection. BS, GW and QT conceived of the study, and participated in design and coordination and helped to draft the manuscript. All authors read and approved the final manuscript.

## Funding

This work was supported by grants from the National Nature Scientific Foundation of China (81770639,82070657), Applied technology research and development project of Heilongjiang Province (GA20C019), Outstanding youth funds of the first affiliated hospital of Harbin Medical University (HYD2020JQ0006) and Research projects of Chinese Research Hospital Association (Y2019FH-DTCC-SB1).

## Acknowledgments

Thanks are due to Hao Li and Rujuan Liu for assistance with statistical analysis in this study.

## Conflict of interest

The authors declare that the research was conducted in the absence of any commercial or financial relationships that could be construed as a potential conflict of interest.

## Publisher’s note

All claims expressed in this article are solely those of the authors and do not necessarily represent those of their affiliated organizations, or those of the publisher, the editors and the reviewers. Any product that may be evaluated in this article, or claim that may be made by its manufacturer, is not guaranteed or endorsed by the publisher.
